# Missense mutation in *SLIT2* associated with congenital myopia, anisometropia, connective tissue abnormalities, and obesity

**DOI:** 10.1186/s13023-018-0885-4

**Published:** 2018-08-15

**Authors:** Katherine Y. Liu, Jesse D. Sengillo, Gabriel Velez, Ruben Jauregui, Lynn Y. Sakai, Irene H. Maumenee, Alexander G. Bassuk, Vinit B. Mahajan, Stephen H. Tsang

**Affiliations:** 1 Stony Brook University School of Medicine, Stony Brook, NY USA; 2 Jonas Children’s Vision Care, and Bernard and Shirlee Brown Glaucoma Laboratory, New York, USA; 3 Department of Ophthalmology, Columbia University, New York, NY USA; 4 Department of Medicine, Reading Hospital, West Reading, PA USA; 5 Omics Laboratory, Stanford University, Palo Alto, CA USA; 6 Department of Ophthalmology, Byers Eye Institute, Stanford University, Palo Alto, CA USA; 7 Medical Scientist Training Program, University of Iowa, Iowa City, IA USA; 8 Weill Cornell Medical College, New York, NY USA; 9 Departments of Molecular and Medical Genetics and Biochemistry and Molecular Biology, Oregon Health and Science University and Shriners Hospital for Children, Portland, USA; 10 Department of Pediatrics, University of Iowa, Iowa City, IA USA; 11 Palo Alto Veterans Administration, Palo Alto, CA USA; 12 Department of Pathology and Cell Biology, Stem Cell Initiative (CSCI), Institute of Human Nutrition, College of Physicians and Surgeons, Columbia University, New York, NY USA; 13 Harkness Eye Institute, Columbia University Medical Center, 635 West 165th Street, Box 212, New York, NY 10032 USA

**Keywords:** SLIT2, Myopia, Anisometropia, Structural modeling, Precision medicine, Obesity

## Abstract

**Background:**

SLIT2 is a protein ligand for the Roundabout (ROBO) receptor and was found to play a major role in repulsive midline axon guidance in central nervous system development. Based on studies utilizing knockout models, it has been postulated that SLIT2 is important for preventing inappropriate axonal routing during mammalian optic chiasm development.

**Methods:**

Case report.

**Results:**

Here, we report a case of congenital myopia, anisometropia, and obesity in a patient with a *SLIT2* point mutation. Examination of the patient’s skin biopsy revealed abnormalities in elastin and collagen fibrils that suggest an underlying connective tissue disorder. Structural modeling placed the novel mutation (p.D1407G) in the EGF-like domain 8 and was predicted to affect interactions with SLIT2 binding partners.

**Conclusions:**

To the authors’ knowledge, this is the first report of a *SLIT2* variant in the context of these ocular findings.

**Electronic supplementary material:**

The online version of this article (10.1186/s13023-018-0885-4) contains supplementary material, which is available to authorized users.

## Background

Myopia is the most common ocular disorder. High-grade myopia is a leading cause of visual impairment and blindness worldwide, particularly due to associated comorbidities that include retinal detachment, localized retinal degeneration, premature cataract and glaucoma. Multiple genetic syndromes with extraocular findings manifest with myopia as a clinical feature, including the autosomal dominant connective tissue disorders Marfan syndrome and Stickler syndromes type 1 and 2, all of which can be traced back to defects in fibrillin, *COL2A1*, and *COL11A1* genes, respectively [1]. Non-syndromic high-grade myopia is frequently early onset and congenital. Congenital myopia is generally regarded as a multi-factorial polygenic disorder. The role of genetic factors in the development of non-syndromic congenital myopia is not clearly understood due to the wide clinical spectrum and genetic heterogeneity of this condition. Multiple twin studies demonstrated evidence of the heritability of myopia, including increased concordance of refractive error and refractive components (axial eye length, corneal curvature, lens power, anterior chamber depth) in monozygotic twins compared with dizygotic twins [2]. The estimated heritability estimates from twin studies range from 0.5 to 0.96. The chance of an individual having myopia if their sibling is affected, expressed as a ratio to the general population, is approximately 4.9 to 19.8 for siblings for high-grade myopia (− 6.00 spherical D or greater), and approximately 1.5 to 3 for low-grade or common myopia (approximately − 1.00 to − 3.00 spherical D), suggesting the presence of genetic risk factors for both high-grade myopia and low-grade myopia [3].

Additionally, multiple genetic loci associated with myopia are identified. The largest linkage scan to date for familial high-grade myopia utilized whole exome sequencing data from 254 families from five independent sites, demonstrating linkage replication of the high myopia loci *MYP1*, *MYP3*, *MYP6, MYP11, MYP12*, and *MYP14*, and identifying a novel locus at chromosome 9q34.11 [3, 4]. Other studies have identified implicated genes. For example, autosomal-recessive high-grade myopia was reported in a large consanguineous Israeli Bedouin kindred. Genetic analysis and sequencing of the exons of six genes identified a point mutation c.1523G > T in the exon 10 of the *LEPREL1* gene [5]. The uromodulin-like 1 (*UMODL1*) gene, which was previously prioritized during a whole-genome case-control association analysis in high-myopia Japanese patients, has been found to have one significant SNP within its frequent recombinant region, supporting the gene’s potential role as a disease susceptibility gene. A case control study of mixed ethnicities showed an association between myopia and 2 SNPs in the collagen 2 alpha 1 gene (*COL2A1*), which maps to chromosome 12q13.11 and has been associated with familial Stickler syndrome type 1. A retrospective analysis of patients with type II collagenopathy chondrodysplasia further revealed that over 85% are myopic, suggesting that myopia may result from defects in type II collagen in these cases [4]. These studies underscore the inherent complexity of myopia and the potential genetic factors contributing to refractive error.

The SLIT2 protein is a major ligand for the Roundabout (ROBO) receptor and was initially found to play a major role in repulsive midline axon guidance in CNS development [6–10]. In humans, SLIT Homolog 2 (*SLIT2*), was mapped to chromosome 4p15.2 [6, 7]. In healthy individuals, *SLIT2* is expressed in a wide variety of tissues, such as connective and adipose tissue, heart, brain, eye, vasculature, and kidney. SLIT/ROBO paired signaling is now known to be critical for a wide variety of morphogenetic processes, such as chemotaxis, angiogenesis, kidney and cardiac development. It has also been shown to impede the pathologic formation of blood vessels [11–15]. SLIT2 is also now known to be important for the establishment of the polarity of newly differentiated retinal ganglion cells (RGC) along the optic pathway [16]. Further, the full-length SLIT2 protein (180 kDa) is cleaved into a 130 kDa N-terminal fragment (termed SLIT2-N) and a 50 kDa C-terminal fragment (SLIT2-C). The SLIT2-C fragment has been found to play a key role in regulating glucose homeostasis and energy expenditure in adipocytes by activating PKA-dependent signaling pathways [17]. Human mutations in *SLIT2* have been previously-identified in patients with congenital abnormalities of the kidney and urinary tract (CAKUT) and recurrent mutations have been detected in patients with small-cell lung cancer [18, 19]. In this case study, we report a novel tetrad of congenital myopia, anisometropia, obesity, and connective tissue abnormalities in a patient with a variant in *SLIT2*, c.4220A > G (p.D1407G).

## Methods

### Phenotypic ascertainment

The patient underwent an ophthalmic examination which included spectral domain-optical coherence tomography (SD-OCT) images and fundus autofluorescence (AF) images, which were acquired using a Spectralis HRA + OCT (Heidelberg Engineering, Heidelberg, Germany). Full field electroretinograms (ffERG) were obtained using the Diagnosys Espion Electrophysiology System (Diagnosys LLC, Littleton, MA, USA) and Ganzfield stimulation per international standards. The pupils were maximally dilated before full-field ERG testing using guttate tropicamide (1%) and phenylephrine hydrochloride (2.5%). Additionally, the corneas were anesthetized with guttate proparacaine 0.5%.

### Genetic testing

Whole exome sequencing, bioinformatics analysis, and filtering based on autosomal and X-linked dominant and recessive and Y-linked inheritance models of the proband, mother, father and maternal aunt were conducted at Ambry Genetics Laboratory. Manual review to rule out sequencing artifacts and polymorphisms along with medical interpretation to rule out genes lacking clinical overlap with the patient’s evaluated phenotype resulted in one candidate gene with likely clinical relevance that was selected for further investigation via co-segregation analysis.

### Structural modeling of human SLIT2

The leucine-rich repeat domains (LRR1–4; residues 30–909) were modelled off the mouse Toll-like receptor-9 structure (PDB: 3WPF; 24% sequence identity) [20]. and the human SLIT2 dimerization domain D4 (PDB: 2WFH; 100% sequence identity) using MODELLER 9.14 [21, 22]. The structure of the EGF-like repeats 1–6 (residues 918–1157) were modelled off the Notch1 crystal structure (PDB: 5UK5; 39% sequence identity) [23]. The structure of the laminin C domain was modeled off the laminin alpha structure (PDB: 1OKQ; 31% sequence identity) [24]. The structure of EGF-like repeats 7–9 were modelled off the Notch1 ligand Delta-like 1 structure (PDB: 4XBM; 98% sequence identity) [25]. There were no homologous structures in the Protein Data Bank for the C-terminal cysteine knot (CTCK) domain. We therefore modeled this domain using an ab initio approach in Phyre2 [26]. The individual domain models were then assembled through ab initio domain assembly using the AIDA program [27]. In silico mutagenesis was performed using FoldX [28]. Electrostatic potentials were calculated using APBS [29]. Protein and solvent dielectric constants were set to 2.0 and 78.0, respectively. PyMOL generated all structural figures [30].

## Results

**Fig. 1:**
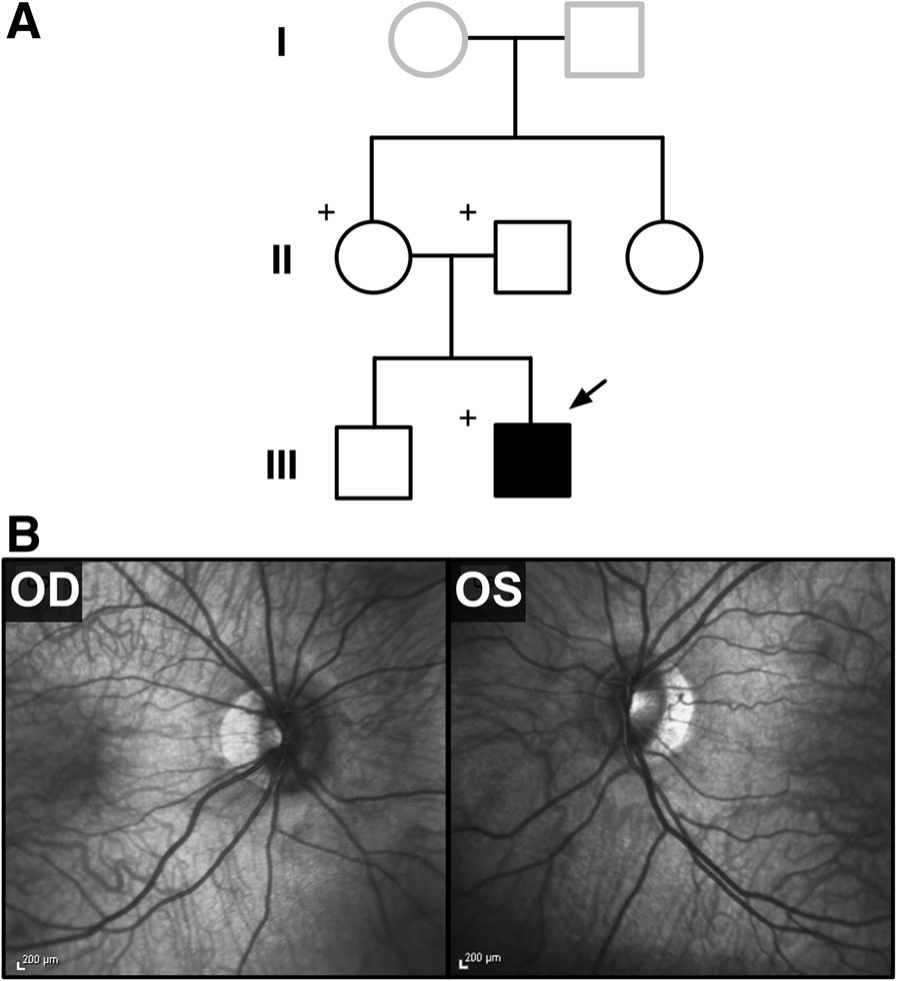
Clinical examination of a patient with myopia, anisometropia, obesity, and connective tissue abnormalities: **a** Pedigree of the proband family. The patient in this family is the only one presenting with ocular disease. The (+) denotes family members who underwent whole exome sequencing. **b** Dilated fundus examination reveals peri-papillary atrophy of the optic nerve, seen also in SD-OCT

A 15-year-old boy presented to the Harkness Eye Institute electroretinography clinic. Initially, best-corrected visual acuity was 20/50 in the right eye and 20/40 in the left eye. His past medical history was significant for obesity and conception through IVF (Additional file 1: Table S1) [31]. Family history was unremarkable. His older brother was unaffected and had no visual complaints (Fig. 1a). Systemic evaluation showed mild joint laxity bilaterally in the upper and lower extremities and mildly doughy skin particularly in the ears. There were no known congenital kidney anomalies reported by history. The proband never achieved 20/20 vision as per history. The proband’s vision was 20/50 in the right eye and 20/40 in the left eye since 18-months-of-age. His mother has been patching the left eye since 18-months-of-age. Yearly follow-up examinations showed no signs of either strabismus or nystagmus. The anterior segment examination appeared to be quiet and without cataracts. The corneas were clear and extraocular eye movements were symmetric and full. On dilated fundus examination, the patient’s optic nerve presented with a good rim and peripapillary atrophy, a common finding in the general population, and is shown on spectral domain optical coherence tomography (SD-OCT) (Fig. 1b). Multifocal electroretinogram testing was performed per ISCEV standards with 61 hexagons. The waveforms were mildly reduced compared to normal and were consistent with macular dysfunction. The axial length of patient’s right eye increased from 26.94 mm in January 2010 to 27.75 mm 3 years later, further increasing to 28.55 mm as measured in his latest clinic visit on January 2017. The axial length of patient’s left eye has increased from 25.67 mm in January 2010 to 26.75 mm 3 years later, further increasing to 27.73 mm on January 2017. Refraction was − 7 sphere with − 4 cylinder at 22 degrees and − 4.25 sphere with − 2.25 cylinder at 142 degrees for the right and left eye, respectively. On examination in January 2017, refraction progressed to − 9 sphere with − 4.75 cylinder at 25 degrees and − 7 sphere with − 3.5 cylinder at 152 degrees for the right and left eye, respectively.

**Fig. 2:**
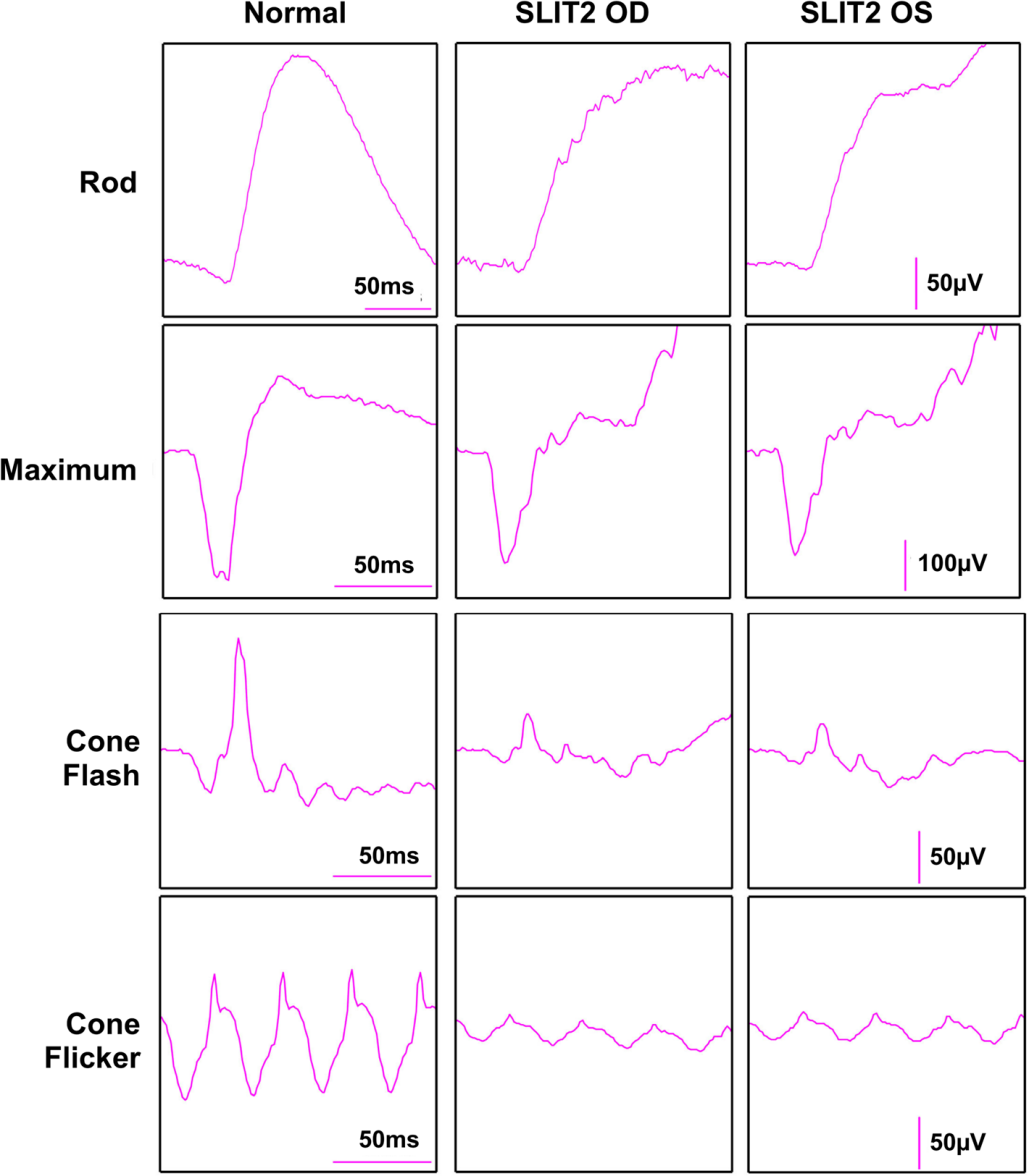
Electroretinogram: Full field electroretinogram results performed using DTL recording electrodes and Ganzfeld stimulation per international standards

Full field electroretinogram (ffERG) testing showed scotopic rod specific ERG b-wave amplitudes were 159 microvolts in the right eye and 156 microvolts in the left eye. Photopic 30 Hz flicker ERG had amplitudes were 25 microvolts in the right eye and 29 microvolts in the left eye. Scotopic and photopic responses exhibited no implicit time delays (Fig. 2). After three-years, the patient’s visual acuity was found to be best corrected to 20/40 in the right eye and 20/30 in the left eye, remaining relatively stable over follow-up.

**Fig. 3:**
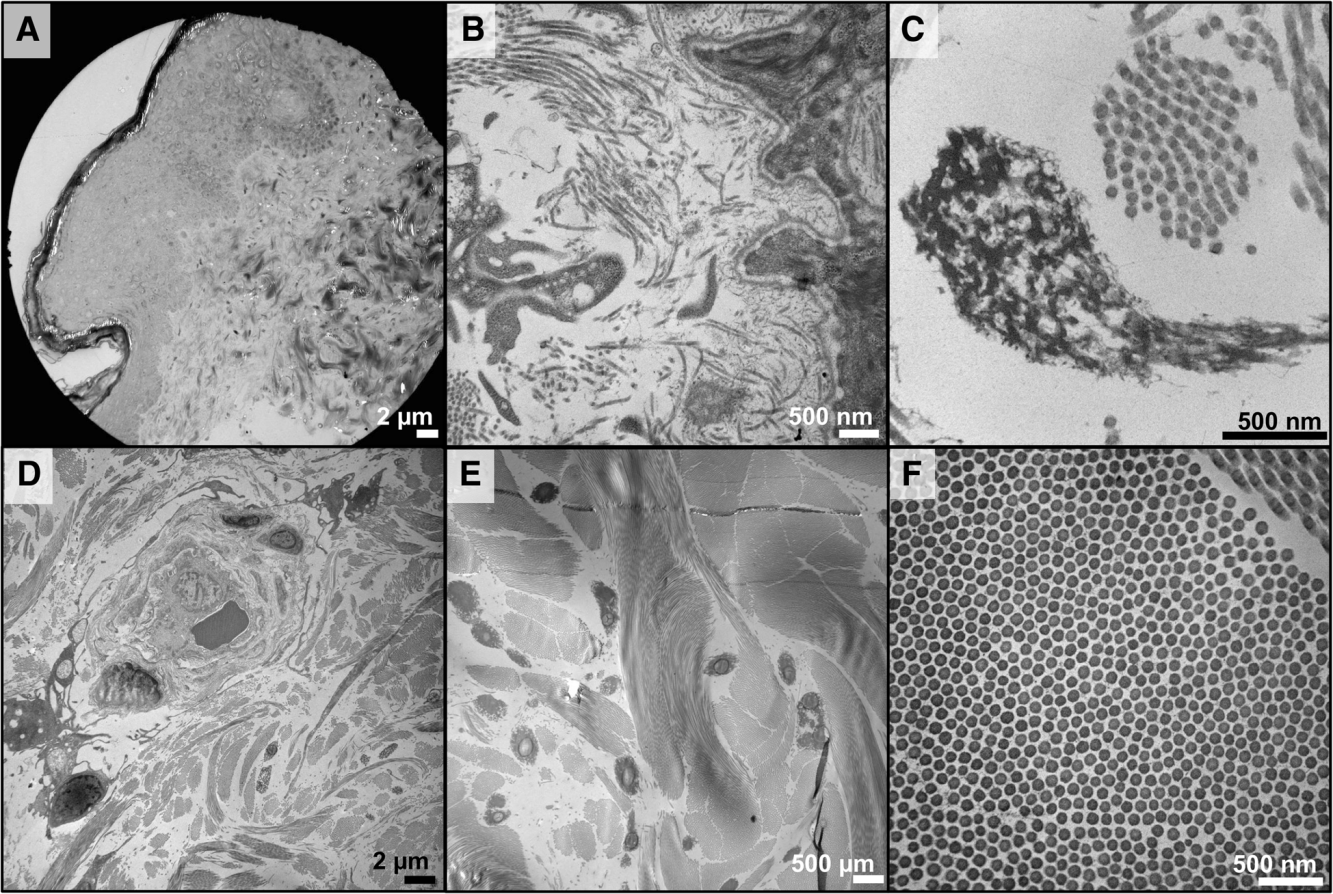
Histological analysis reveals connective tissue abnormalities: **a** Skin biopsy showing considerably thickened epithelium, particularly near the hair follicles. Image taken at 220× magnification; scale bars = 10 μm. **b** Skin biopsy showing high density of collagen in the papillary dermis. Image taken at 19000× magnification; scale bars = 500 nm. **c** Skin biopsy showing elastin in the shallow and deep papillary dermis that is moth eaten and lacks associated micro-fibrils. Image taken at 50000× magnification; scale bars = 500 nm. **d** Skin biopsy showing macrophages adjacent to the capillaries. Image taken at 3500× magnification; scale bars = 2 μm. **e** Skin biopsy showing dimples at the cores of the elastin fibrils that seem to indicate a higher than normal density of elastin fibrils in the reticular dermis. Image taken at 3500× magnification; scale bars = 2 μm. **f** Skin biopsy showing collagen fibrils in the reticular dermis are abnormally small and uniform in diameter. Image taken at 29000× magnification; scale bars = 500 nm

A skin biopsy was performed and examined by light microscopy. Examination by light microscopy revealed multiple abnormalities. A considerably thickened epithelium was present, particularly near the hair follicles (Fig. 3a), along with a high density of collagen in the papillary dermis (Fig. 3b). Furthermore, there are large deposits of microfibrils adjacent to the basement membrane, which is often seen in tissue that is repeatedly injured. The elastin in the shallow and deep papillary dermis is moth-eaten and lacks associated microfibrils, which would not be expected in a child (Fig. 3c). The biopsy also showed macrophages adjacent to the capillaries (Fig. 3d) and dimples at the cores of the elastin fibrils that indicated a higher than normal density of elastin fibrils in the reticular dermis (Fig. 3e). The collagen fibrils in the reticular dermis were also abnormally small and uniform in diameter (Fig. 3f).

Whole exome sequencing was performed on the peripheral blood of the proband and his family members, including parents, brother and maternal aunt. No coding variants were identified in known myopia, Bardet-Biedl syndrome or retinitis pigmentosa genes. There was a novel heterozygous variant identified in exon 36 of the *SLIT2* gene of the proband, c.4220A > G, p.D1407G. The unaffected mother, father, brother and maternal aunt did not carry this mutation, indicating a likely de novo occurrence in the patient (Additional file 1: Tables S2-S5). A primary sequence analysis in SIFT [32] and PolyPhen-2 [33] predicted the mutation to be tolerated, while PROVEAN [34] predicted deleterious effects on SLIT2 function (Additional file 1: Tables S6-S8).

**Fig. 4:**
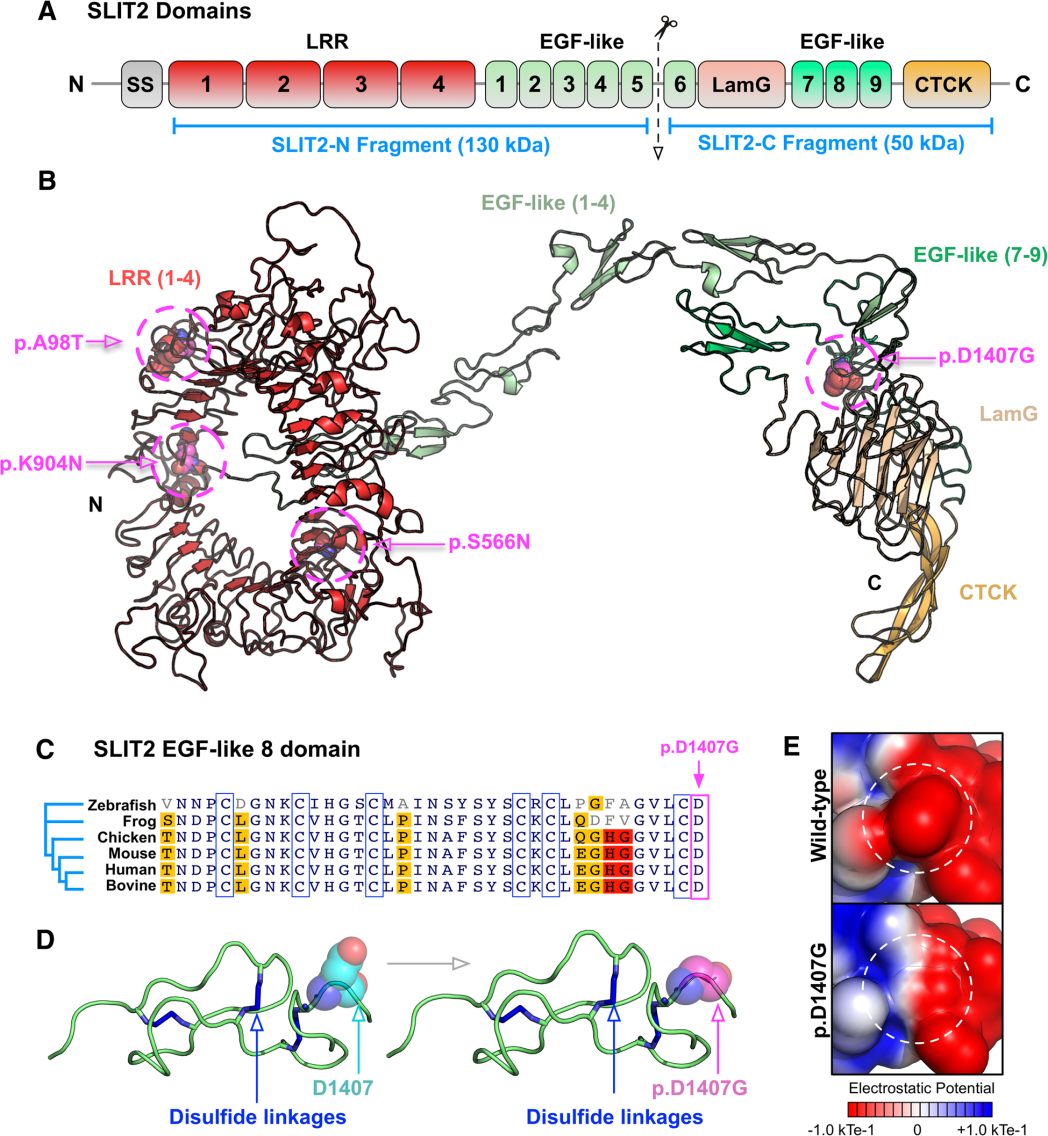
Structural modeling of patient SLIT2 mutation: **a** Diagram of SLIT2 domains. The SLIT2 protein is processed into two fragments, SLIT2-N and SLIT2-C. The approximate site of proteolytic cleavage is shown. **b** Structural model of the full-length human SLIT2 protein generated through a domain assembly approach (see Additional file 1). The p.D1407G mutation is located in the SLIT-C region in the EGF-like domain 8. **c** Multiple sequence alignment of SLIT2 EGF-like 8 domains from multiple species reveals conservation of the D1407 residue. EGF-like domains contain six conserved cysteine residues (highlighted by the blue outline) that form three disulfide bridges that provide structural rigidity to the domain. **d** The D1407 residue is located adjacent to a disulfide bridge which would restrain the residue. Substitution with glycine would lead to more conformational flexibility at this site and potentially destabilize the domain by altering disulfide bond formation. **e** In addition to destabilizing the EGF-like domain, the p.D1407G mutation disrupts a negative charge at this site. Electrostatic surface potentials calculated using APBS software highlight the loss of negative charge in this region, which may be critical for maintaining interactions with SLIT2-C binding partners

We performed computer-based structural modeling to gain insight into the pathogenicity of our patient’s SLIT2 mutation [35–37]. *SLIT2* gene encodes a 1529 amino acid extracellular protein which contains no transmembrane sequence [6, 7]. All SLIT proteins share a common structure which includes an N-terminal signal peptide (SS), four tandem leucine-rich repeats (LRR), a sequence of EGF repeats, a conserved ALPS spacer (laminin G) followed by a C-terminal cysteine knot (CTCK), which serves as a dimerization motif (Fig. 4a) [6, 7]. Structures of several human SLIT2 domains have been solved by x-ray crystallography, but the full-length structure remains to be determined [9, 21, 38]. We therefore generated a three-dimensional model of the full-length SLIT2 structure using a domain assembly approach [39] (Fig. 4b; Additional file 1). Our structural model placed the p.D1407G mutation on the SLIT2-C fragment in the 8th EGF-like repeat domain. Previously-identified CAKUT mutations (A98T, S566 N, and K904 N) were all located on the LRR domains of the SLIT-N fragment [19]. The D1407 amino acid was shown to be highly conserved throughout vertebrates, consistent with the evolutionary conservation of exon 36 measured in PhyloP [40] (Additional file 1: Table S9). This finding suggested that mutation of this amino acid from aspartate to a different amino acid would not be well tolerated and likely lead to negative effects, such as those observed in the patient (Fig. 4c). Further analysis showed that the D1407 residue is restrained by an adjacent cysteine-disulfide bridge, one of three highly-conserved disulfide linkages in this domain (Fig. 4d). The p.D1407G mutation is predicted to cause a decrease in stability (∆∆G of − 0.35 kcal/mol), likely due to the increased conformational flexibility of the substituted glycine (Fig. 4d) [28]. Additionally, this substitution leads to a loss of negative charge (Fig. 4e). Since EGF-like domains are known to be involved in mediating protein-protein interactions, this loss of charge may affect interactions to SLIT2 binding partners that are critical to its molecular function [41, 42].

## Discussion

In this case we present a 15-year old boy with a tetrad of congenital myopia, anisometropia, obesity, and connective tissue abnormalities. The patient’s skin biopsy indicated an absence in the coordination of elastic fiber formation and the integration of elastin fibers with collagen fibers, suggesting an underlying connective tissue disorder. Whole exome sequencing revealed a novel heterozygous variant in exon 36 of the *SLIT2* gene of the proband, c.4220A > G, p.D1407G. The heterozygous nature of the variant associated with the patient’s phenotype suggests, most likely, a potential gain of function in the expressed protein, although paternity was not tested in the pedigree. Based on data from the NHLBI Exome Sequencing Project (ESP), the c.2240A > G alteration in *SLIT2* was not observed among 6503 individuals tested (0.0%) (assessed December, 2017). Furthermore, the D1407 amino acid was shown to be highly conserved throughout vertebrates, meaning that evolution of this amino acid from aspartate to a different amino acid is likely not well tolerated and leads to negative effects.

It is now known that the SLIT2/ROBO4 paired signaling impedes the pathologic formation of blood vessels and reduces vascular leakage in mouse models [43]. These pathological processes are hallmarks of age-related macular degeneration, premature retinopathy, and diabetic retinopathy. The SLIT2/ROBO receptor signaling also helps to guide retinal ganglion cell axons to extend into the optic fiber layer in the dorsal periphery of the retina and project toward the optic disc [44]. Furthermore, it has been shown that Slit binds to type IV Collagen, and that this interaction stabilizes the Slit molecule in the basement membrane at the surface of the tectum, contributing to the retinotectal architecture. Collagen IV appears to organize the cellular scaffold at the surface of the tectum, which consists of radial glial end feet and secreted factors such as Slit, which together, serve as laminar positional cues to ingrowing retinal axons [45]. It is well established that myopia can be caused by increased axial length of the eye and pathologic changes in the sclera, including scleral thinning, particularly in the posterior pole of the eye. The sclera is a dense connective tissue that maintains the eye shape and is comprised mainly of extracellular matrix which is primarily made up of collagen. In the development of myopia, there is a significant loss of scleral tissue weight that is associated with a narrowing and disconnection of collagen fiber bundles and a reduction in the number of them, especially at the posterior pole. As myopia continues to develop, the thinning of existing collagen fiber bundles is accompanied by a shift in collagen fiber diameter distribution such that the sclera contains more small collagen fibers, which accounts for the lower tensile strength of the tissue [46]. These pathologic findings related to collagen in myopia development, along with the findings in this case of the *SLIT2* mutation and abnormal collagen in the patient’s skin biopsy, suggest a potential role of the *SLIT2* mutation in defective connective tissue formation, indicating a more systemic genetic syndrome that encompasses the patient’s congenital myopia.

The long-term systemic consequences of *SLIT2* c.4220A > G, p.D1407G in connective tissue, the heart, brain, eye, vasculature, and the kidney are unknown. *SLIT2* is found in the extracellular matrix of these organs. A single mutation in another extracellular matrix protein, fibrillin, initially causes myopia in children and then predisposes systemic complications later in life [1]. Structural modeling of this mutation has provided some initial insight into its pathogenicity. Our analysis suggests that this mutation destabilizes SLIT2 interactions with its binding partners by disrupting a highly conserved residue in the EGF-like domain 8. This mutation is distinct from previously-published CAKUT mutations (which are located in the LRR domains) and offer a potential explanation for the tissue specificity of their related phenotypes [19].

## Conclusions

This case provides compelling evidence of the *SLIT2* point mutation as a novel gene associated with the identified ocular findings and connective tissue abnormalities. These conclusions are limited, as they are derived from a singular case thus far, and there exists the possibility that there are other unidentified variants contributing to aspects of the patient’s phenotype, such as obesity, that may be unrelated to the *SLIT2* mutation [47]. This further underscores the importance of future studies to shed light on the role of *SLIT2* in connective tissue pathophysiology, obesity, and ocular disease.

## Additional file

Additional file 1: **Table S1.** BMI observations for proband and brother. **Table S2.** *SLIT2* candidate gene. **Table S3.** *SLIT2* mutation whole exome sequencing results. **Table S4.** Familial genetic co-segregation analysis by whole exome sequencing. **Table S5.** Variant filtering based on bioinformatics and interpretation. **Table S6.** Prediction of effects of SLIT2 mutations using the SIFT server. **Table S7.** Prediction of effects of SLIT2 mutations using the PolyPhen-2 server. **Table S8.** Prediction of effects of SLIT2 mutations using the PROVEAN server. **Table S9.** PhyloP conservation scores for *SLIT2*. (DOCX 35 kb)

## Abbreviations

AF: Autofluorescence
CAKUT: Congenital abnormalities of the kidney and urinary tract
COL2A1: Collagen 2 alpha 1 gene
CTCK: C-terminal cysteine knot
ESP: Exome sequencing project
ffERG: Full-field electroretinogram
ROBO: Roundabout receptor
SD-OCT: Spectral domain optical coherence tomography
UMODL1: Uromodulin-like 1 gene
