# Supplementary material for: Missense mutation in SLIT2 associated with congenital myopia, anisometropia, connective tissue abnormalities, and obesity
Source: Orphanet J Rare Dis. 2018 Aug 15;13:138. doi: 10.1186/s13023-018-0885-4 (PMC6094464; doi:10.1186/s13023-018-0885-4)
Supplement: Supplementary file 1 — Table S1. BMI observations for proband and brother. Table S2. SLIT2 candidate gene. Table S3. SLIT2 mutation whole exome sequencing results. Table S4. Familial genetic co-segregation analysis by whole exome sequencing. Table S5. Variant filtering based on bioinformatics and interpretation. Table S6. Prediction of effects of SLIT2 mutations using the SIFT server. Table S7. Prediction of effects of SLIT2 mutations using the PolyPhen-2 server. Table S8. Prediction of effects of SLIT2 mutations using the PROVEAN server. Table S9. PhyloP conservation scores for SLIT2. (DOCX 35 kb) [file 13023_2018_885_MOESM1_ESM.docx]

**Supplementary Online Content**

**Missense mutation in *SLIT2* associated with congenital myopia, anisometropia, connective tissue abnormalities, and obesity**

Katherine Y. Liu^2^, Jesse D. Sengillo^3,4^, Gabriel Velez^5,6,7^, Ruben Jauregui^3,8^, Alexander G. Bassuk^9^, Vinit B. Mahajan^5,6,10^, Stephen H. Tsang^1,3,11§^

^1^Jonas Children’s Vision Care, and Bernard & Shirlee Brown Glaucoma Laboratory

^2^Stony Brook University School of Medicine, Stony Brook, NY, USA

^3^Department of Ophthalmology, Columbia University, New York, NY, USA

^4^State University of New York Downstate Medical Center, Brooklyn, NY, USA

^5^Omics Laboratory, Stanford University, Palo Alto, CA

^6^Department of Ophthalmology, Byers Eye Institute, Stanford University, Palo Alto, CA

^7^Medical Scientist Training Program, University of Iowa, Iowa City, IA

^8^Weill Cornell Medical College, NY, USA

^9^Department of Pediatrics, University of Iowa, Iowa City, IA

^10^Palo Alto Veterans Administration, Palo Alto, CA

^11^Department of Pathology & Cell Biology, Stem Cell Initiative (CSCI), Institute of Human Nutrition, College of Physicians and Surgeons, Columbia University, New York, NY, USA.

^§^Corresponding author

Running title: *SLIT2* mutation and associated clinical findings

Address Correspondence:

Stephen H. Tsang, MD, PhD

Harkness Eye Institute

635 West 165th Street, Box 212, New York, NY 10032

Phone: (212) 342-1189 / Fax: 212-305-4987 / Email: gene.editing@gmail.com

**Supplemental Table 1.** BMI observations for proband and brother – Page 2

**Supplemental Table 2.** *SLIT2* candidate gene – Page 3

**Supplemental Table 3.** *SLIT2* mutation whole exome sequencing results – Page 4

**Supplemental Table 4.** Familial genetic co-segregation analysis by whole exome sequencing – Page 5

**Supplemental Table 5.** Variant filtering based on bioinformatics and interpretation – Page 6

**Supplemental Table 6.** Prediction of effects of SLIT2 mutations using the SIFT server – Page 7

**Supplemental Table 7.** Prediction of effects of SLIT2 mutations using the PolyPhen-2 server – Page 8

**Supplemental Table 8.** Prediction of effects of SLIT2 mutations using the PROVEAN server – Page 9

**Supplemental Table 9.** PhyloP conservation scores for *SLIT2* – Page 10

**Supplemental Table 1.** Body habitus descriptions for proband and brother.

|  | **Proband** | **Brother** |
| --- | --- | --- |
| **Age** | **Body Habitus** | **Body Habitus** |
| 5 | Overweight-appearing | Normal-appearing |
| 10 | Obese-appearing | Normal-appearing |
| 12 | Obese-appearing | Normal-appearing |

**Supplemental Table 2.** *SLIT2* candidate gene.

| **Gene** | **Location** | **Protein** | **Genomic coordinates (GRCh37)** | **Genomic Size (bp)** | **RefSeq#** | **Total Exons** | **Coding Exons** |
| --- | --- | --- | --- | --- | --- | --- | --- |
| *SLIT2* | 4p15.2 | Slit homolog 2 (Drosophila) | Chr4:20255235-20620788 | 365,554 | NM_004787 | 37 | 37 |

**Supplemental Table 3.** *SLIT2* mutation whole exome sequencing results.

| **Gene** | **Characterized/Novel** | **Protein Change** | **Nucleotide Change** | **Genotype** | **Alteration Type** | **Alteration** | **Gene Overlap** |
| --- | --- | --- | --- | --- | --- | --- | --- |
| *SLIT2* | Novel | p.D1407G | c.4220A>G | Heterozygous | Missense | Uncertain | Uncertain |

**Supplemental Table 4.** Familial genetic co-segregation analysis by whole exome sequencing.

| **Gene** | **Filtering Model** | **Alteration Location** | **Alteration** | **Proband 12-60838** | **Brother 12-60843** | **Father**  **12-60844** | **Mother 12-60846** | **Aunt 12-64586** |
| --- | --- | --- | --- | --- | --- | --- | --- | --- |
| *SLIT2* | AD | Exon 36 | c.4220A>G  p.D1407G | +/- | -/- | -/- | -/- | -/- |

**Supplemental Table 5.** Variant filtering based on bioinformatics and interpretation.

| **Genes (Alterations)** | **Post Inheritance Model Filtering** | **Post-Medical Review** | | | | **Candidate Genes** |
| --- | --- | --- | --- | --- | --- | --- |
|  |  | **Post-Alteration Review TOTAL** | **Post-Clinical Association Review** | | |  |
|  |  |  | **Characterized** | **Clinically Novel** | **TOTAL** |  |
| **Autosomal Dominant** | 14(16) | 5(5) | 0(0) | 5(5) | 5(5) | 1(1) |
| **Autosomal Recessive** | 7(17) | 2(4) | 0(0) | 1(2) | 1(2) | 0(0) |
| **X-Linked Recessive** | 5(5) | 4(4) | 0(0) | 4(4) | 4(4) | 0(0) |
| **X-Linked Dominant** | 0(0) | 0(0) | 0(0) | 0(0) | 0(0) | 0(0) |
| **Y-Linked** | 0(0) | 0(0) | 0(0) | 0(0) | 0(0) | 0(0) |
| **Totals** | 26(38) | 11(13) | 0(0) | 10(11) | 10(11) | 1(1) |

1. **Definition of Variant Filtering Table provided by AmbryGenetics Whole Exome Sequencing:**
2. **(A)  Post-medical review:** Medical review filtering involves the manual removal of genes unrelated to the patient's evaluated phenotype and alterations considered benign.
3. **(B)  Post-alteration review:** The remaining variants (novel + characterized) remaining after removal of polymorphisms and sequencing artifacts.
4. **(C)  Post-clinical association review:** The number of remaining genes after removing those with insufficient clinical overlap based on the patient’s phenotype. The remaining variants represent i) clinically novel genes about which there is insufficient knowledge to rule out and ii) characterized genes with at least a minimal level of phenotypic overlap.
5. **(D)  Candidate Alterations:** Genes with consistent phenotypic overlap (both “uncertain” and positive findings). Candidate genes undergo co-segregation analysis either via automated fluorescence dideoxy sequencing (aka “Sanger”) confirmation or via trio exome sequencing data with Q- score and read depth above established confidence thresholds.
6. **(E)  Characterized post-clinical association genes which are not candidate alterations:** Genes included here represent characterized genes with at least a minimal level of phenotypic overlap. If a gene is listed in this column but was not selected as a candidate gene, the gene is categorized as an “Unlikely Clinically Significant Finding” (UCSF) and is described in a table within the report’s supplemental information along with an explanation for the characterization.

**Supplemental Table 6.** Prediction of effects of SLIT2 mutations using the SIFT server.

| **Substitution** | **Prediction** | **Score** | **Median Info** | **Number of sequences at position** |
| --- | --- | --- | --- | --- |
| A98T | Tolerated | 0.06 | 2.93 | 133 |
| S566N | Damaging | 0.04 | 2.57 | 165 |
| K904N | Tolerated | 0.28 | 2.31 | 182 |
| **D1407G** | **Tolerated** | **0.09** | **2.30** | **191** |

**Supplemental Table 7.** Prediction of effects of SLIT2 mutations using the PolyPhen-2 server.

| **Substitution** | **Prediction** | **HumDiv Score** |
| --- | --- | --- |
| A98T | Probably Damaging | 0.992 |
| S566N | Probably Damaging | 0.984 |
| K904N | Possibly Damaging | 0.738 |
| **D1407G** | **Benign** | **0.166** |

**Supplemental Table 8.** Prediction of effects of SLIT2 mutations using the PROVEAN server.

| **Substitution** | **Prediction** | **PROVEAN Score** |
| --- | --- | --- |
| A98T | Deleterious | 0.003 |
| S566N | Neutral | 0.012 |
| K904N | Neutral | 0.113 |
| **D1407G** | **Deleterious** | **0.006** |

**Supplemental Table 9.** PhyloP conservation scores for *SLIT2*.

| **Gene** | **Chrom** | **Data Start** | **Data End** | **Minimum** | **Maximum** | **Range** | **Mean** | **Variance** | **Standard Deviation** |
| --- | --- | --- | --- | --- | --- | --- | --- | --- | --- |
| *SLIT2* | chr4 | 20618768 | 20619009 | -4.714 | 10.003 | 14.717 | 4.52997 | 14.4353 | 3.79938 |
